# Fabrication of initial trabecular bone-inspired three-dimensional structure with cell membrane nano fragments

**DOI:** 10.1093/rb/rbac088

**Published:** 2022-11-02

**Authors:** Koichi Kadoya, Emilio Satoshi Hara, Masahiro Okada, Yu Yang Jiao, Takayoshi Nakano, Akira Sasaki, Takuya Matsumoto

**Affiliations:** Department of Biomaterials, Okayama University Graduate School of Medicine, Dentistry and Pharmaceutical Sciences, Okayama 700-8558, Japan; Department of Maxillofacial Surgery, Okayama University Graduate School of Medicine, Dentistry and Pharmaceutical Sciences, Okayama 700-8558, Japan; Department of Biomaterials, Okayama University Graduate School of Medicine, Dentistry and Pharmaceutical Sciences, Okayama 700-8558, Japan; Department of Biomaterials, Okayama University Graduate School of Medicine, Dentistry and Pharmaceutical Sciences, Okayama 700-8558, Japan; Department of Biomaterials, Okayama University Graduate School of Medicine, Dentistry and Pharmaceutical Sciences, Okayama 700-8558, Japan; Division of Materials & Manufacturing Science, Osaka University, Osaka 565-0871, Japan; Department of Maxillofacial Surgery, Okayama University Graduate School of Medicine, Dentistry and Pharmaceutical Sciences, Okayama 700-8558, Japan; Department of Biomaterials, Okayama University Graduate School of Medicine, Dentistry and Pharmaceutical Sciences, Okayama 700-8558, Japan

**Keywords:** trabecular bone, calcospherites, cell membrane nano fragments, three dimensionalization, bone tissue synthesis

## Abstract

The extracellular matrix of trabecular bone has a large surface exposed to the bone marrow and plays important roles such as hematopoietic stem cell niche formation and maintenance. *In vitro* reproduction of trabecular bone microenvironment would be valuable not only for developing a functional scaffold for bone marrow tissue engineering but also for understanding its biological functions. Herein, we analyzed and reproduced the initial stages of trabecular bone formation in mouse femur epiphysis. We identified that the trabecular bone formation progressed through the following steps: (i) partial rupture of hypertrophic chondrocytes; (ii) calcospherite formation on cell membrane nano fragments (CNFs) derived from the ruptured cells; and (iii) calcospherite growth and fusion to form the initial three-dimensional (3D) structure of trabecular bones. For reproducing the initial trabecular bone formation *in vitro*, we collected CNFs from cultured cells and used as nucleation sites for biomimetic calcospherite formation. Strikingly, almost the same 3D structure of the initial trabecular bone could be obtained *in vitro* by using additional CNFs as a binder to fuse biomimetic calcospherites.

## Graphical Abstract

**Figure:**
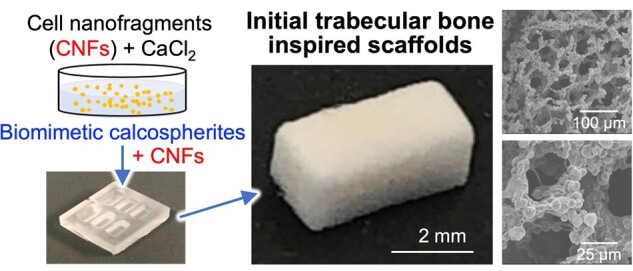

## Introduction

Bone tissues have critical roles in body support, organ protection, metabolism and hematopoiesis. So far, the understanding of molecular and cellular mechanisms involved in bone formation and structure have been widely investigated [1–4]. Especially, the trabecular bone has attracted a great attention in recent decades because it is also important for the formation and maintenance of the stem cell niche [5–9]. For example, previous studies indicated that hematopoietic stem cell (HSC) niche was often found near the trabecular bone (e.g. in the trabecular-rich metaphysis of long bones) [6], and was supported by mesenchymal stem cells (MSCs), bone metabolism-related osteoblasts and osteoclasts and vascular endothelial cells [5–7]. However, the understanding of trabecular bone formation, as well as bone marrow formation, is still poorly understood. Of note, *in vitro* reproduction of trabecular bones would be valuable not only as model systems to investigate the cell-to-cell and cell-to-matrix interactions related to the stem cell niche, but also as a functional scaffold for bone marrow tissue engineering.

There are numerous studies applying biomaterials, cells and functional molecules to fabricate trabecular bone-like three-dimensional (3D) structures *in vitro* for bone tissue engineering [10]. However, most of the previous studies have not been intended for reproducing the trabecular bone functions and bone marrow tissues. Besides, since the composition and structure of scaffolds affect the cellular functions and behaviors [11, 12], fabrication of trabecular bone-like scaffolds with highly mimicking the native tissue would be critically important to fabricate and regenerate trabecular bone with bone marrow tissues.

For designing functional scaffolds, it would be valuable to observe/analyze the tissue formation and development processes *in vivo*. We have been analyzing the bone tissue formation process in femur epiphysis [13], and found that hypertrophic chondrocytes showed intracellular vacuolar degeneration and ruptured (i.e. burst) in the femur epiphysis of newborn mice at postnatal Day 5 (P5). The hypertrophic chondrocyte rupture was shown to be triggered by external mechanical (e.g. force) and chemical (e.g. hypotonic and alkaline) stimuli [14, 15]. By the cell rupture, parts of the cell membrane (i.e. nanofragments), which was tightly bound to the extracellular matrix (ECM) through integrins, were torn and remained attached with the ECM. We also found that the residual cell membrane nano fragments (CNFs) acted as the nucleation site for the formation of calcospherite—a calcified globule consisting of hydroxyapatite (HAp) [16, 17]. We proposed the CNFs-induced mineral formation is one of the initial endochondral ossification mechanisms, as which the matrix vesicle-mediated mineral formation is widely regarded [18, 19]. However, the development mechanism of how the initially formed calcospherites transform into a 3D trabecular bone (i.e. how calcospherites fuse to each other to form a porous trabecular bone) remains largely obscure.

Therefore, in this study, we first performed a more detailed observation of the initial trabecular bone formation and development process. Based on the results, we tried to reproduce the trabecular bone formation *in vitro* using culture cell-derived CNFs as the core material.

## Materials and methods

### Observation of initial trabecular bone formation and development process in mouse femur epiphysis

Animal experiments were performed according to the Guidelines for Animal Research of Okayama University, under the approval of the Animal Care and Use Committee of Okayama University (OKU-2017076 and OKU-2020287).

BALB/c mice from postnatal Day 6 (P6) to P12 (Shimizu Laboratory Supplies, Kyoto, Japan) were sacrificed by an overdose of isoflurane and the femurs were dissected. The femur epiphysis was then isolated with a scalpel and immersed in a 12% NaClO solution for 24 h to remove organic components. The NaClO-treated sample was washed with ultrapure water, dehydrated with ethanol, replaced with butanol and freeze-dried. The conductive treatment of samples was performed with an osmium coater (Neoc-Pro, Meiwafosis Co. Ltd., Tokyo, Japan) at an electrical discharge current of 10 mA and a degree of vacuum of 10 Pa for 20 s. Secondary electron images were observed with a field-emission scanning electron microscope (FE-SEM: JSM-6701F, JEOL Ltd., Tokyo, Japan) at 5 kV and 10 mA. For backscattered electron observation with a FE-SEM, ion-milled sections of resin-embedded bones without NaClO treatments were prepared based on the protocol described previously [17].

The X-ray diffraction (XRD: Rint 2500HF, Rigaku Corp., Tokyo, Japan) analysis and the energy dispersive X-ray spectroscopy (EDS: EDAX ApolloXP, Mahwah, NJ, USA) were, respectively, carried out for crystallographic and elemental analysis of the samples. This study was approved by the Animal Care and Use Committee of Okayama University (OKU-2017076 and OKU-2020287).

### Preparation of CNFs

ATDC5 cells (RIKEN BRC, Ibaragi, Japan), a mouse chondrocytic progenitor cell, were cultured in Dulbecco’s Modified Eagle Medium/F12 (FUJIFILM Wako Pure Chemical Corp., Osaka, Japan) containing 10% fetal bovine serum (Life Technologies, MD, USA) until confluency. The cells were detached from the culture dishes by using trypsin-EDTA solution (FUJIFILM Wako Pure Chemical Corp.) and collected in 50 ml tubes. The cells were then resuspended in distilled water, at a concentration of 1.0 × 10^7^ cells/ml. The suspension containing 1.0 × 10^7^ cells was transferred into 1.5 ml tubes and disrupted by an ultrasonic homogenizer (Handy Sonic UR-20P, Tomy Seiko Co. Ltd., Tokyo, Japan) for 3 min. Our previous study has shown that 3-min ultrasonic disruption was the most appropriate method for obtaining uniform CNFs [16]. After the ultrasonic homogenization, centrifugation (15 000 rpm, 20 min) was performed to remove organelles.

### *In vitro* fabrication of biomimetic calcospherites with CNFs

The optimal conditions for the *in vitro* calcospherite formation by CNF mineralization were examined. After the CNFs suspended in distilled water containing *β*-glycerophosphate (*β*-GP, Sigma-Aldrich, St. Louis, MO, USA) were transferred into a 60-mm dish, a 200-µl calcium chloride solution (0–20 mM; pH 9; FUJIFILM Wako Pure Chemical Corp.) was added, and then the mixture was incubated for 3 days. The obtained calcospherites were centrifugally washed, dropped onto an aluminum holder and dried in vacuum. The calcospherites were then coated with osmium for SEM observation (S-4800, Hitachi High-Tech Corp., Tokyo, Japan) available at the Central Research Laboratory, Okayama University Medical School. The XRD analysis was also carried out to analyze the sample’s characteristics.

### Affinity between HAp and CNFs

To evaluate the adsorption behavior, the CNF suspension (10 µl) was dropped onto a HAp plate (10 × 10 × 1 mm^3^; HOYA Corp., Tokyo, Japan) and after washing with distilled water and NaCl solutions with higher ionic strengths (1%), the remained molecules on the HAp plate were determined by using an attenuated total reflection Fourier transform infrared spectrophotometer (IRAffinity-1S, Shimadzu Corp., Kyoto, Japan). To evaluate the adhesion strength, the CNF suspensions (0–1000 mg/ml) were placed between two HAp plates and dried at 37°C for 6 h. The adhesion strength between two HAp plates was measured using a universal mechanical tester (EZ test, Shimadzu Corp.). Since the fatty acid is one of the major components of cellular membrane, sodium oleate (FUJIFILM Wako Pure Chemical Corp.) was also used for the adsorption (10 mg/ml) and adhesion (0–200 mg/ml) experiments as described above.

### Reproduction of initial trabecular bone-inspired scaffolds

The 30 µl of biomimetic calcospherites suspension were mixed with or without CNF suspension (5 µl) and dropped in a rectangular 3D-printed mold (4 × 2 × 2 mm^3^). The mold was frozen at –20°C, and then freeze-dried in vacuum. The dried sample was fixed onto an aluminum holder, coated with osmium and observed by SEM.

## Results

### Observation of initial trabecular bone formation and development process in mouse femur epiphysis

**Figure 1.**
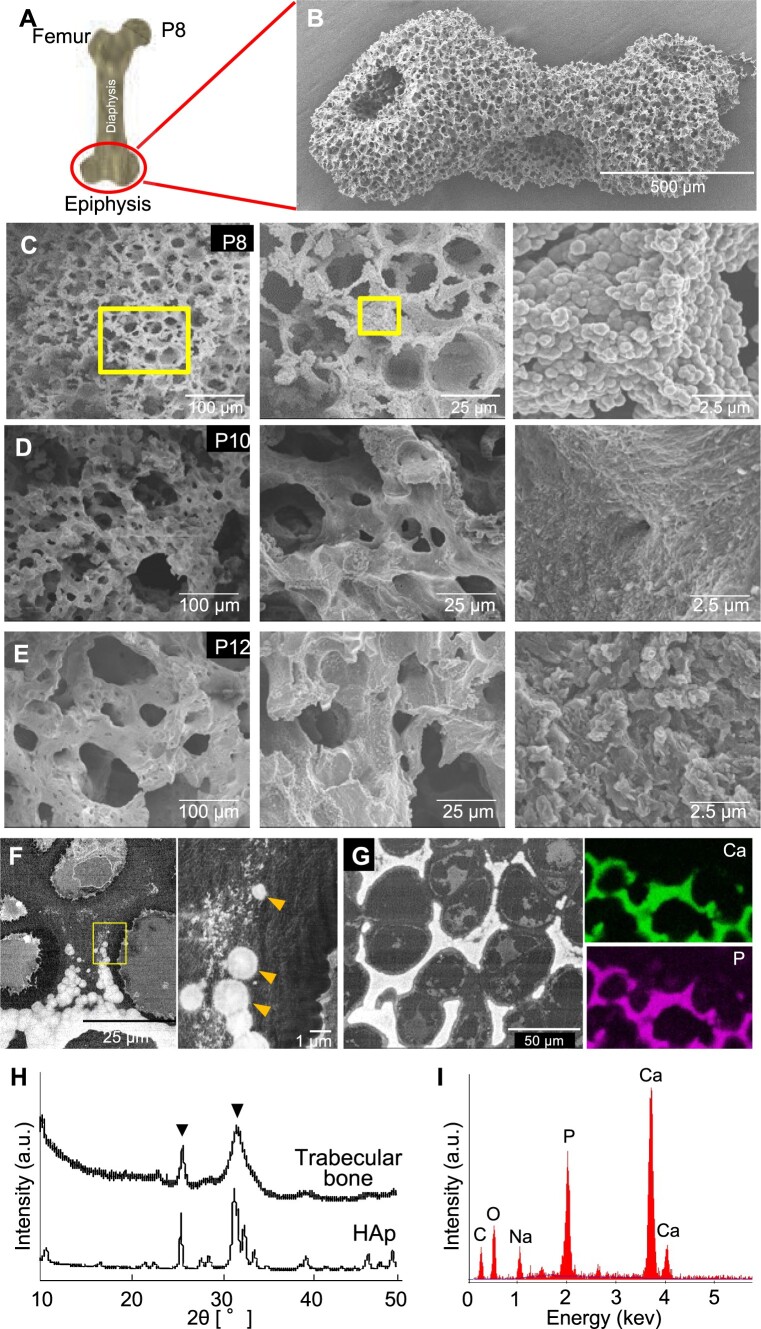
Analysis of the initial stages of trabecular bone formation in mouse femur epiphysis. (**A**) Schematic design of mouse femur. Circle indicates the epiphysis. (**B**) Secondary electron image of the initially formed trabecular bone at postnatal Day 8 (P8) after NaClO treatment for removal of all organic components. (**C–E**) Secondary electron images of the trabecular bones at (C) P8, (D) P10 and (E) P12 observed at different magnifications. (**F**) and (**G**) Backscattered electron images of a cross section near the front of the trabecular bone at P8. Right panel in (F) shows a higher magnified image of the area within the yellow squares in the left panel, and arrowheads indicate the calcospherites. Whitish multiform materials in the vicinity of the calcospherites would be rich in osmium-stained lipids of cell membrane fragments [14]. In the left panel in (G) taken at lower magnification, the fusion of calcospherites could be observed on the right upper area. Note that after calcospherite fusion, the trabecular bone became compact (left down area). Right panels in (G) indicate the localization of Ca and P in the trabecular bone. (**H**) XRD analysis showing the low crystalline HAp in the initially formed trabecular bone at P8. Commercially available HAp was used as a reference. (**I**) EDS analysis showing high peaks for Ca and P in the native trabecular bone at P8.

The initial trabecular bone formed in the epiphysis of the extracted mouse femur was observed by SEM. Our previous study showed that mineralization initiated at this site at P5.5 [14]. The trabecular bone (after removal of organic components by NaClO treatment) at P8 showed a 3D porous structure as shown in Fig. 1A and B. A higher magnified observation revealed that the pore surface consisted of a lot of fused calcospherites (Fig. 1C). Backscattered electron images (without removal of organic components) indicate that the pores were formed because of calcospherite formation in the surroundings of hypertrophic chondrocytes (Fig. 1F). The higher levels of Ca and P were clearly observed in the trabecular bone, as detected by elemental mappings (Fig. 1G). The quantitative EDS analysis showed that the trabecular bone contained large amounts of Ca and P, but a small amount of C (Fig. 1I). XRD analysis confirmed that the calcium phosphate minerals in the initial trabecular bone was low crystalline HAp (Fig. 1H).

At P10 (Fig. 1D) and P12 (Fig. 1E), the borders of the globule-like structures observed at P8 became blurred. The unevenness of the wall surface decreased gradually, and the trabecular bone became overall denser. The pore size also increased compared to that at P8 (Fig. 1C–E).

### *In vitro* fabrication of biomimetic calcospherites with CNFs

**Figure 2.**
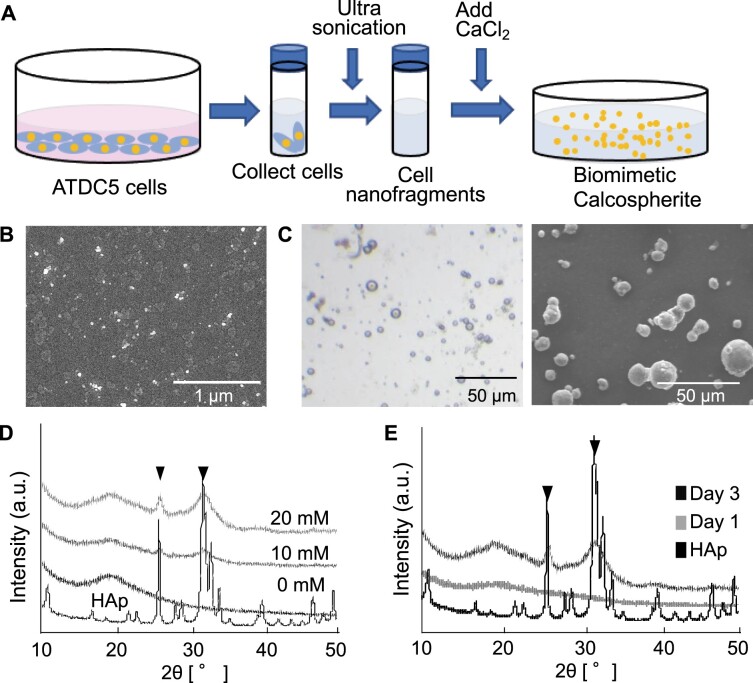
*In vitro* fabrication of calcospherites with cell nanofragments (CNFs). (**A**) Schematic design showing the protocol for fabrication of biomimetic calcospherites. ATDC5 chondrogenic cells were cultured until confluency and after being collected in 1.5 ml tubes, they were fragmented by ultrasonication for 3 min to obtain CNFs. The CNFs were mixed with a CaCl_2_ solution and incubated for 3 days to fabricate calcospherites. (**B**) An SEM image of CNFs collected from the cells. (**C**) An optical micrograph (left panel) and an SEM image (right panel) of the biomimetic calcospherites incubated for 3 days. (**D**) XRD analysis showing the characteristics of the biomimetic calcospherites prepared with different concentrations of CaCl_2_. Note the peaks corresponding with those of HAp in the samples incubated with 10 and 20 mM CaCl_2_ solutions. (**E**) XRD analysis of the biomimetic calcospherites after incubation for 1 or 3 days in 20 mM CaCl_2_ solution. Note the peaks matching those of HAp in the calcospherites incubated after 3 days. Commercially available HAp was used as a reference.

Our previous works indicated that the CNFs, which were derived from the partial rupture of hypertrophic chondrocytes in the femur epiphysis and remained in the matrix between the chondrocytes, induced the formation of calcospherites [14]. To reproduce these phenomena and to induce biomimetic calcospherite formation *in vitro*, CNFs derived from cultured chondrocytes were used as nucleation site (Fig. 2A and B). The formation of small minerals was observed after 1 day incubation with the calcium chloride solution containing *β*-GP, which would be hydrolyzed by phosphatases anchored to CNFs and act as a phosphate ion source [15, 16], and the size of the product increased after 3-day incubation. Of note, the morphology of the product (Fig. 2C) was closely resembling the calcospherites observed in the femur epiphysis (Fig. 1C). XRD analysis revealed the formation of low crystalline HAp in the sample supplemented with 20 mM Ca solution (Fig. 2D). Of note, XRD analysis also revealed that in the sample with 20 mM Ca solution, there was an initial formation of amorphous calcium phosphate, which transformed into HAp after 3-day incubation, as shown in Fig. 2E.

### Affinity between HAp and CNFs

**Figure 3.**
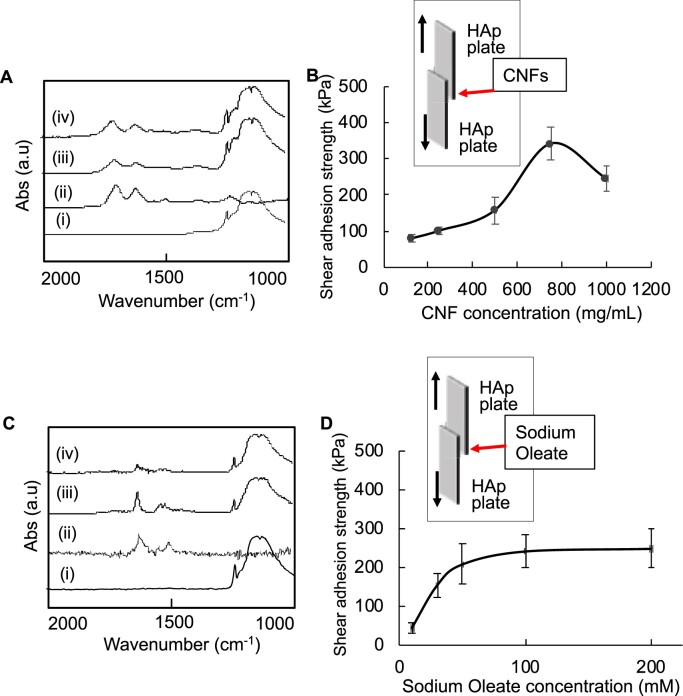
Affinity tests between hydroxyapatite and organic molecules. (**A**) and (**C**) FT-IR analyses for the affinity tests of absorbents (A: CNFs and B: oleic acid) on a HAp plate: (i) intact HAp plate; (ii) intact adsorbent; (iii) HAp plate after dropping the adsorbent solution; (iv) HAp plate after washing with sodium chloride solutions. (**B**) and (**D**) Shear adhesion strengths of HAp plates bound with different concentrations of (B) CNFs (125, 250, 500, 750 and 1000 mg/ml) and (D) sodium oleate (10, 30, 50, 100 and 200 mM).

CNFs were derived from cell membrane and therefore those consisted of lipids and macromolecules (i.e. proteins, glycoproteins and nucleic acids). We hypothesized that CNFs would act not only as a nucleation site for calcospherite formation but also as a binder of calcospherites. To examine this hypothesis, we investigated the affinity of CNFs on commercially available HAp plates qualitatively by using a flushing test with high ionic strength solution. As a result, high affinity of CNFs to HAp was confirmed because the CNFs adsorbed on HAp did not desorb completely even after washing at a high ionic strength (Fig. 3A). Next, CNF suspension was applied to bond two HAp plates and shear adhesion strength was evaluated. As shown in Fig. 3B, there was almost a linear increase in the shear adhesion strength between two HAp plates in a concentration-dependent manner up to 750 mg/ml of CNFs. The adhesion strength increased further reaching a peak of 341 kPa at 750 mg/ml, and subsequently decreased to 245 kPa at a concentration of 1000 mg/ml of CNFs. Since CNFs contain large amounts of fatty acids in the plasma membrane, we also evaluated the affinity of sodium oleate HAp. Sodium oleate remained after the flush test at a high ionic strength (Fig. 3C), and the adhesive strength increased dose-dependently reaching 248 kPa at a concentration of 200 mM (Fig. 3D).

These results suggested that the possibility of CNFs as a binder of calcospherite fusion in the trabecular bone formation.

### Reproduction of initial trabecular bone-inspired scaffolds

**Figure 4.**
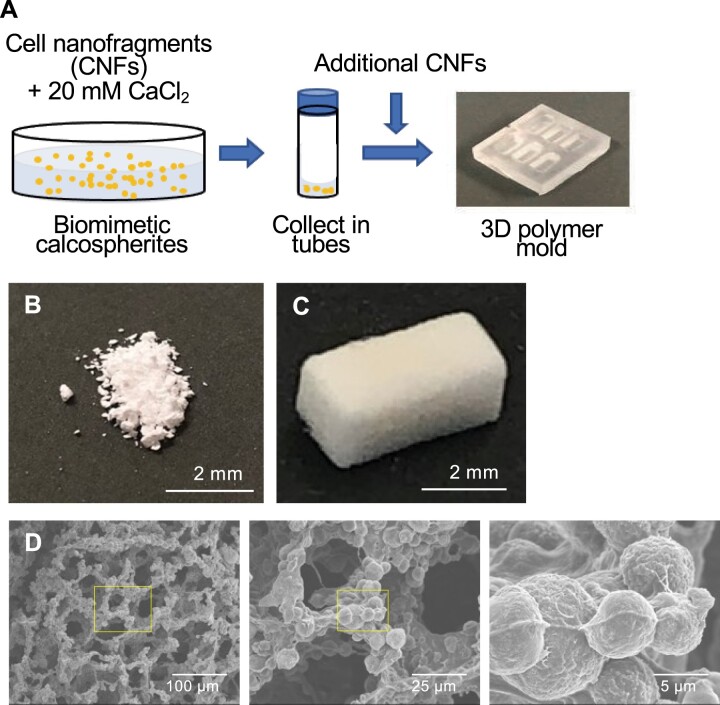
*In vitro* fabrication of a 3D trabecular bone-like construct. (**A**) Schematic design showing the protocol for fabrication of the 3D trabecular bone-like construct. CNFs were mixed and incubated with 20 mM CaCl_2_ solution for 3 days for fabrication of biomimetic calcospherites, which were centrifugally washed and collected in tubes. The calcospherites were further mixed with CNFs and dropped into a 3D-printed mold. (**B**) and (**C**) Digital photographs of the biomimetic calcospherites incubated (B) without and (C) with additional CNFs inside the 3D printed mold. A 3D structure could only be obtained with CNFs to the previously formed biomimetic calcospherites. (**D**) Secondary electron images of the 3D trabecular bone-like construct showing similar architecture and porosity compared to those of the native trabecular bone. Note that the fusion of calcospherites is observed in the right panel.

Next, we attempted to reproduce a 3D trabecular bone-like scaffolds consisting of the biomimetic calcospherites as a building block and additional CNFs as a binder. The calcospherite–CNFs mixture was dropped into a rectangular mold and freeze-dried (Fig. 4A). As expected, in the group without additional CNF binders, 3D porous materials could not be obtained, and calcospherite powders remained in the mold (Fig. 4B). On the other hand, the calcospherite–CNF mixture allowed the formation of a 3D structure (Fig. 4C), and its porous structure was similar to the initial trabecular bone *in vivo*. At a higher magnification, it was confirmed that the calcospherites fused to form the wall surface of this porous 3D structure (Fig. 4D) similar to the pore surfaces of trabecular bone at P8 (Fig. 1C).

## Discussion

The bone marrow formation in developing long bones involve various processes, such as the invasion of nestin^+^ MSCs and CD31^+^ endothelial cells that forms the blood vessels, and migration of HSCs into the cartilaginous tissue where the initial trabecular bone has been formed [20]. Here, the trabecular bone is considered as a crucial site for homing and further maintenance of the HSC niche [21]. Addition to this, trabecular bone is recognized as a crucial site to modulate cancer cell metastasis [8, 22, 23]. Although there are numbers of studies indicating the involvement of diverse cells and chemokines, there is not much discussion about the effect of the structure, size and composition of the trabecular bone on the formation of bone marrow or cancer metastasis [24–26]. Thus, we herein focused on reproducing the trabecular bone via bottom-up approaches using CNFs to highly mimic the *in vivo* process of trabecular bone formation.

SEM observations revealed that the initial trabecular bone at the femur epiphysis showed a porous 3D structure at P8. The walls of this 3D structure were formed by fusing calcospherites, and the pores reflect the site where hypertrophic chondrocytes were originally present. In previous studies, we have shown that the CNFs formed by the rupture of hypertrophic chondrocytes were the nucleation sites for *in vivo* mineralization [17]. Therefore, it is suggested that initial trabecular bone formation proceeds through the following steps: (i) partial rupture of hypertrophic chondrocytes; (ii) calcospherites formation from the cell membrane fragments derived from the ruptured cells; and (iii) calcospherites growth and fusion around the ruptured cells to form the 3D structure of trabecular bones *in vivo*. As discussed below, we also hypothesized that CNFs would not be only for a nucleation site of mineral formation but also a binder for the calcospherite fusion. Other proteins (including fibrous Types II and X collagens or long-chain proteoglycans) abundantly present in the region of initial trabecular bone formation [27] would also contribute to the calcospherite fusion and mechanical properties of the developing trabecular bone.

Based on the observations *in vivo*, we attempted to reproduce the initial stage of trabecular bone tissue formation *in vitro* by using CNFs collected from cultured chondrogenic cells. CNFs consists of lipids and macromolecules (i.e. proteins, glycoproteins and nucleic acids) and previous reports have demonstrated that nucleic acids induce mineralization [28, 29], which would be due to the cleavage of the phosphate groups by phosphatases for the release of free phosphate ions that subsequently react with calcium, and form calcium phosphate minerals [30]. Our previous study demonstrated that the plasma membrane fraction could be the nucleation site for mineral formation *in vitro* [15, 17] by isolating only the plasma membrane fraction from cultured cells [31]. Additionally, phosphoproteins in the plasma can also be strong candidates participating in the mineralization process of the cell nanofragments. By mixing CNFs with calcium solution under optimized conditions, we succeeded in forming mineralized globules with almost the same shape as those of the calcospherites observed in the initially formed trabecular bone. Moreover, the results indicated that the calcospherite size changes according to the incubation time. Since the calcospherites size differs between endochondral and membranous ossification [17, 32], this method would be valuable to control the calcospherite size and modulate the mechanical properties of final products.

Construction of a 3D trabecular bone-like structure mimicking the *in vivo* findings is of great important for detailed investigations at cellular and subcellular levels. To date, porous 3D scaffolds have been widely fabricated in bone tissue engineering. Methods for fabricating a porous scaffold structure include salt leaching, firing and freeze-drying [33–36]. Since the CNFs contain large number of proteins, freeze-drying method was herein used to maintain their original functions. When attempting to construct the 3D trabecular bone-like structure, *in vitro* fabricated calcospherites could not bind to each other and remained as powder. Interestingly, however, when these calcospherites were mixed with additional CNFs, a final porous 3D structure was firmly achieved. Therefore, we hypothesized that CNFs would not be only for a nucleation site of mineral formation but also a binder for the calcospherite fusion. In order to examine this hypothesis, the affinity and binding strength of CNFs to HAp were examined. The results support CNFs could be also act as a binder of calcospherite fusion. CNFs consists of lipids and macromolecules (i.e. proteins, glycoproteins and nucleic acids), and we revealed that the lipids could act as a binder of calcospherites *in vitro*. The macromolecules in CNFs are also expected to act as a binder because these macromolecules can adsorb strongly on HAp surfaces [37–41]. Future studies evaluating the interactions between each macromolecular component and HAp are required to clarify the contribution of each component on calcospherite binding.

Several materials have already been reported for reproducing a trabecular bone-like structure. However, these materials are mostly synthetic and not consisted of or containing cell-derived organics. For example, reports indicated that a gelatin hydrogel-based synthetic scaffold manages and guides the functions of HSCs [42, 43]. In this study, we used cells as source materials, and succeeded in forming a 3D trabecular bone-like structure *in vitro*. The fabricated material closely resembled the native trabecular bone in terms of 3D structure in different hierarchies. This 3D trabecular bone-like construct made *via* a bottom-up approach and mimicking not only the architecture, composition and properties, but also the developmental process of the native trabecular bone is a promising scaffold material to future investigations involving HSC niche and cancer metastasis.

## Conclusions

We succeeded in fabricating the initial trabecular bone-like scaffolds by analyzing initial trabecular bone formation *in vivo*. We suggested that CNFs would play critical roles as a binder of the initially formed minerals and promote the 3D architectural development of the trabecular bone. This 3D trabecular bone-like construct would be valuable not only as a bone marrow tissue engineering scaffolds but also as a model for manipulation and understanding of the hematopoietic system.
